# Direct Visualization of De novo Lipogenesis in Single Living Cells

**DOI:** 10.1038/srep06807

**Published:** 2014-10-29

**Authors:** Junjie Li, Ji-Xin Cheng

**Affiliations:** 1 Weldon School of Biomedical Engineering, Purdue University, West Lafayette, IN 47907; 2 Department of Biological Sciences, Purdue University, West Lafayette, IN 47907; 3 Center for Cancer Research, Purdue University, West Lafayette, IN 47907

## Abstract

Increased *de novo* lipogenesis is being increasingly recognized as a hallmark of cancer. Despite recent advances in fluorescence microscopy, autoradiography and mass spectrometry, direct observation of *de novo* lipogenesis in living systems remains to be challenging. Here, by coupling stimulated Raman scattering (SRS) microscopy with isotope labeled glucose, we were able to trace the dynamic metabolism of glucose in single living cells with high spatial-temporal resolution. As the first direct visualization, we observed that glucose was largely utilized for lipid synthesis in pancreatic cancer cells, which occurs at a much lower rate in immortalized normal pancreatic epithelial cells. By inhibition of glycolysis and fatty acid synthase (FAS), the key enzyme for fatty acid synthesis, we confirmed the deuterium labeled lipids in cancer cells were from *de novo* lipid synthesis. Interestingly, we also found that prostate cancer cells exhibit relatively lower level of *de novo* lipogenesis, but higher fatty acid uptake compared to pancreatic cancer cells. Together, our results demonstrate a valuable tool to study dynamic lipid metabolism in cancer and other disorders.

Reprogramming of metabolism in cancers has been known since 1920s^1^. Given Otto Warburg's observation, aerobic glycolysis once was considered as the most prominent change of cancer metabolism. Based on advances in cancer metabolism research in the past decades, current concept of cancer metabolism holds that in addition to aerobic glycolysis, cancer cells also reprogram their mitochondria to satisfy the need for macro-molecular synthesis, instead of maximizing the efficiency of ATP production^2^. One evidence of the reprogrammed mitochondrial metabolism is the increased lipogenesis that has been observed in many cancers.

In mammalian cells, glucose is utilized as the most important energy source and provides precursors for *de novo* synthesis of other macromolecules, like lipids. After glycolysis, pyruvate is produced from glucose and then enters mitochondria. Through the tricarboxylic acid cycle (TCA), pyruvate is converted to acetyl-CoA and exported to cytosol. Acetyl-CoA in the cytosol is used as precursors for lipid synthesis via the *de novo* lipogenesis pathway. Newly synthesized fatty acids are further esterified and stored in lipid droplets (LDs). On the other hand, fatty acids from lysed triglyceride or directly uptake can also be stored in lipids droplets (Fig. 1a)^3^,^4^. Compared to fatty acid uptake, *de novo* lipogenesis seems to play a more important role in many cancers. Overexpression of lipogenic enzymes, including ATP citrate lyase (ACL)^5^, fatty acid synthase (FAS)^6^ and stearoyl-CoA desaturase (SCD)^7^ has been widely reported in many types of cancers. More recent studies showed that under hypoxia condition or in cells with defected mitochondria, glutamine, instead of glucose, mediated lipogenesis becomes more important for tumor growth^8^,^9^. These findings provides valuable insights to the complexity of cancer metabolism. However, it also raised a need to study metabolism in a temporal and spatial resolved manner, especially considering the varieties of microenvironments *in vivo*.

To monitor glucose metabolism, several glucose analogs have been synthesized to mimic the behavior of glucose, e.g., 3-O-methylglucose (3MG), 2-deoxy-D-glucose (2-DG) and fluoro-deoxyglucose (FDG)^10^. Fluorescent labeled glucose analog, 2-[N-(7-nitrobenz-2-oxa-1,3-diazol-4-yl)amino]-2-deoxy-D-glucose (2-NBDG), has been used to study the glucose uptake^11^,^12^. Isotope labeled glucose analog, ^18^F-deoxyglucose (FDG), has been successfully used in clinic to image to tumors in vivo by positron emission tomography (PET)^13^,^14^. However, these analogs are nonmetabolizable and can only be used to study the glucose uptake. Recently, mass spectrometry has been used combined with isotope labeling to study the dynamic process^8^,^9^,^15^,^16^ Although mass spectrometry has high species resolution, it doesn't offer the temporal-spatial resolution to monitor glucose metabolism in single live cells.

Here, we report direct imaging of *de novo* lipid synthesis in single living cells using stimulated Raman scattering (SRS) microscopy that allows real-time vibrational imaging of live cells^17^,^18^,^19^. Earlier, SRS imaging of C–D vibration has been demonstrated to be successful in imaging the incorporation of isotope labeled amino acid into proteins^20^, map drug delivery into skin^21^,^22^ and intracellular fate of fatty acids^23^. More recent advances in coupling small vibration alkyne tags with SRS microscopy opens new opportunities for biological imaging with superb sensitivity^24^,^25^. In this work, we employed SRS microscopy to visualize metabolic reprograming of deuterium-labeled glucose in individual living cells with deuterium-labeled glucose. Unlike the fluorescent analogs or FDG, deuterium substitution does not vary the structure of glucose, nor its physiological functions. With high spatial-temporal resolution of SRS microscopy, we monitored the dynamic metabolism of glucose in single living cancer cells. As the first direct visualization, we observed that deuterium labeled glucose was largely utilized for lipid synthesis in cancer cells, which occurs at a much lower rate in normal immortalized epithelial cells. This method of imaging dynamic metabolism in single living cells could be a valuable tool for elucidating the complexity of the metabolic network and exploring new therapeutic targets for cancer.

## Results

### Imaging *de novo* lipogenesis in living cells

**Figure 1:**
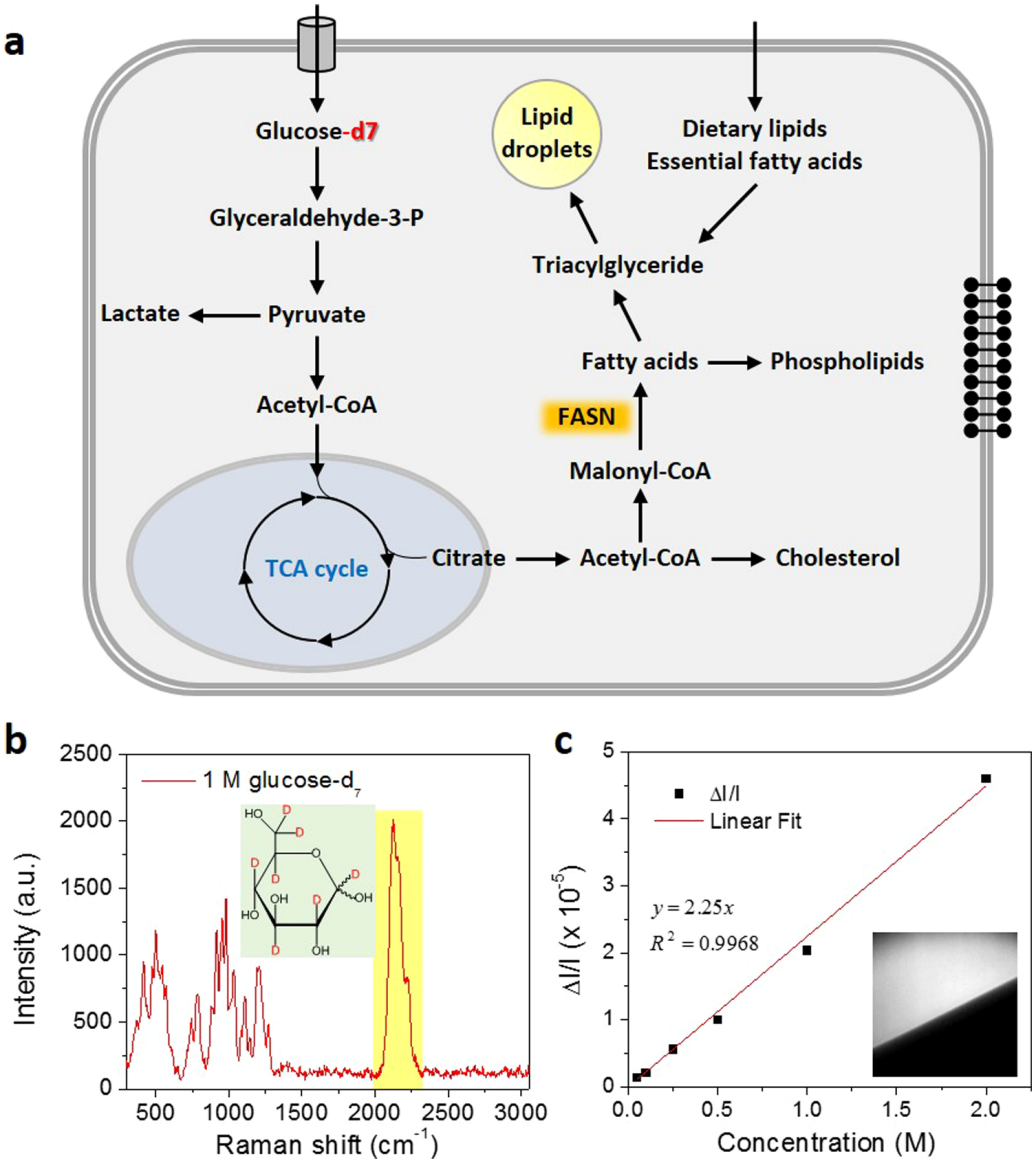
Isotope labeled glucose-d_7_ is a tracer for de-novo lipogenesis. (a). Scheme of glucose-derived de-novo lipid synthesis in mammalian cells. (b). Raman spectrum of 1.0 M glucose-d_7_ aqueous solution. A broad and unique peak around 2120 cm^−1^ from C–D bond vibration was observed. Inset: chemical structure of glucose-d_7_. (c). Linear dependence of SRS signal on glucose-d_7_ concentration. The detection limit of our SRS imaging is around 50 mM. Inset: SRS image of 1.0 M glucose-d_7_ solution at the interface of solution and air.

In order to visualize lipogenesis in living cells, we use a deuterium labeled glucose-d_7_ where the hydrogen atoms are replaced with deuterium atoms (Fig. 1b inset). Raman spectrum taken from a 1.0 M glucose-d_7_ aqueous solution showed a peak centered at ~2120 cm^−1^ from the C–D bond vibration modes (Fig. 1b). This peak resides in the silent region of the Raman spectrum of endogenous molecules, which provides excellent opportunities for SRS imaging of target molecules. By tuning the wavenumber difference between the pump and Stokes beams (ω_p_ − ω_S_) to match the C–D vibration at 2120 cm^−1^, SRS imaging of glucose-d_7_ solution shows a linear dependence of SRS signal on glucose-d_7_ concentration (Fig. 1c), with a detection sensitivity of 50 mM in water.

**Figure 2:**
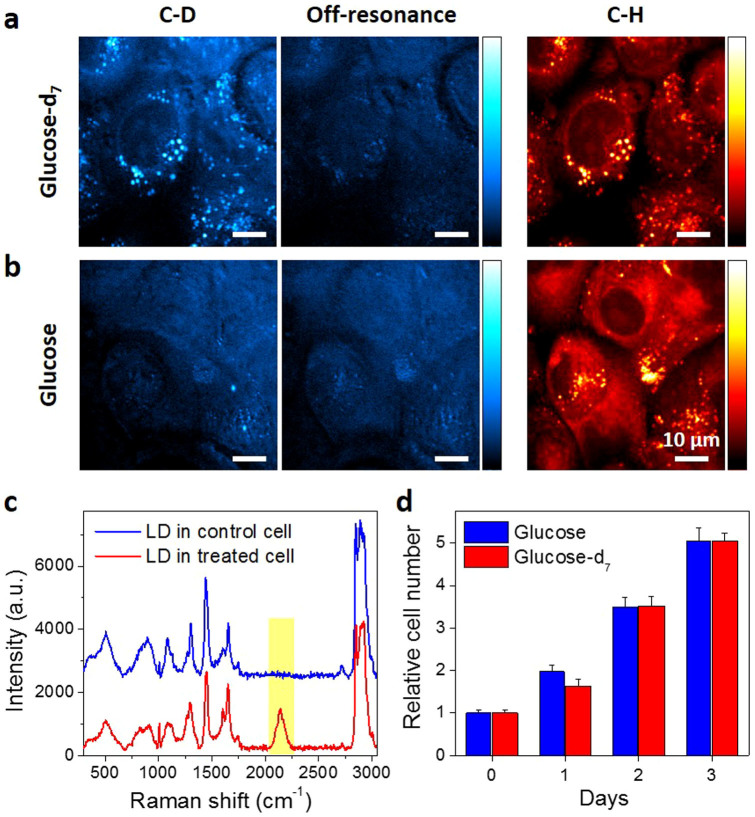
Glucose-d_7_ is utilized for lipid synthesis in pancreatic cancer PANC1 cells. PANC1 cells were incubated with (a). 25 mM glucose-d_7_ or (b). 25 mM glucose in glucose-free DMEM media supplemented with 10% FBS for 3 days. SRS imaging was taken at C–D vibration (~2120 cm^−1^), off-resonance (~2600 cm^−1^) or C–H vibration (~2850 cm^−1^). (c). Raman spectra acquired from lipid droplets in control cells or glucose-d_7_ treated cells. (d). Nontoxicity test of glucose-d_7_ compared to regular glucose. Cell viability was determined by MTT assay. Data was represented as Mean + SD with n = 6 for each group.

To study glucose metabolism inside living cells, we replaced glucose in the normal cell culture medium to deuterium labeled glucose by adding glucose-d_7_ to glucose-free DMEM medium to same concentration (25 mM). PANC1 cells, a pancreatic cancer cell line, were incubated in the medium for 3 days and imaged by SRS microscopy. By matching ω_p_ − ω_S_ to the C–D bond vibration at 2120 cm^−1^, a strong SRS signal was observed in LDs (Fig. 2a, left), which are confirmed by SRS image of lipids at 2850 cm^−1^ (Fig. 2a, right). A video of the *de novo* synthesized lipids showed intracellular trafficking of the LDs (supplementary movie). By tuning (ω_p_ − ω_S_) to 2600 cm^−1^, the off-resonant images showed some weak background (Fig. 2a, middle), which is known to result from cross-phase modulation^26^. As a control, no C–D labeled droplets were detected in the cells cultured with regular high-glucose DMEM medium (Fig. 2b). Raman spectra taken from the LDs in cells further confirmed the presence of C–D bonds in the cell treated with glucose-d_7_, but not in the control cells (Fig. 2c). Collectively, our images and Raman spectra demonstrated that glucose was utilized in pancreatic cancer cells for lipogenesis and excess newly synthesized lipids were stored in LDs. Furthermore, the viability test showed that glucose-d_7_ shows no toxicity to the cells (Fig. 2d). Thus, isotope stably labeled glucose could be used as a tracer to visualize *de novo* lipogenesis in living cancer cells.

### Dynamics of lipogenesis in living cells

**Figure 3:**
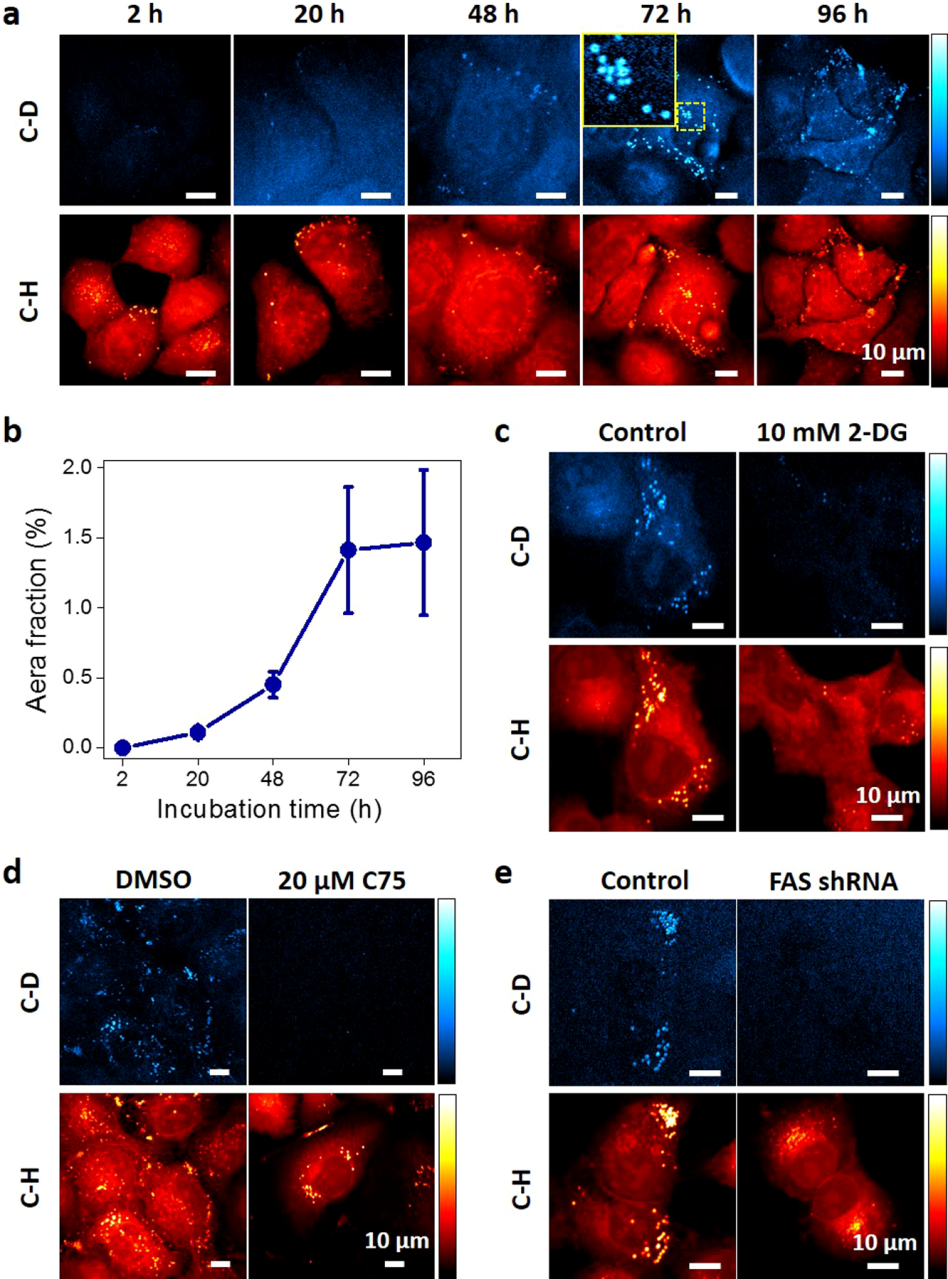
Dynamics of glucose-d_7_ derived lipogenesis in PANC1 cells. (a). Monitoring de-novo lipogenesis in PANC1 cells over time by SRS imaging at C–D and C–H vibration. Inset of image at 72 h: zoom in image of marked area showing the donut-shaped newly synthesized LDs. (b). Quantification of SRS signal at C–D vibration per cell. Data were shown as Mean ± SD. N = 5 for each group. (c). SRS imaging of PANC1 cells incubated with 10 mM 2-DG and 25 mM glucose-d_7_ for 3 days. (d). SRS imaging of PANC1 cells incubated with glucose-d_7_ for 3 days and treated with DMSO or 20 μM FAS inhibitor C75 for 24 hours. (e). SRS imaging of PANC1 cells stably transfected with FAS shRNA and incubated with glucose-d_7_ for 3 days.

The noninvasive property of SRS microscopy enabled us to monitor the dynamic lipogenesis process within the same plate of cells over time. Figure 3a–b showed that the level of *de novo* synthesized lipids kept increasing from 2 to 72 h and then maintained at a steady level after 3-day incubation. The donut shaped LDs were observed (Fig. 3a, inset of 72 h), indicating the newly synthesized lipids deposited at the outer layer of existing LDs. Longer incubation did not further increase the storage of newly synthesized lipids, indicating that lipid storage and lipolysis reached a balanced status after 3 days. To further confirm the deuterium labeled lipids are derived from glucose metabolism pathway, we used an inhibitor of glycolysis, 2-Deoxy-D-glucose (2-DG). With inhibition of glycolysis, we observed reduced number of deuterium labeled LDs (Fig. 3c). Furthermore, to validate the newly synthesized lipids are from *de novo* lipogenesis pathway but not dietary lipids, an inhibitor of fatty acid synthase (FAS), C75, was applied to PANC1 cells. As a result, C75 dramatically reduced the number and intensity of newly synthesized LDs (Fig. 3d). The effect of C75 was further confirmed by knock-down of FAS in PANC1 cells with shRNA (Fig. 3e). Collectively, we verified that the deuterium labeled lipids were derived from glucose metabolism and synthesized through *de novo* lipogenesis pathway. In addition to glucose, glutamine plays as another important precursor for lipogenesis in cancer cells especially under certain conditions (e.g., hypoxia or defected mitochondria)^8^,^9^. To demonstrate the capability our method for broad applications, we also imaged deuterium labeled glutamine derived lipogenesis. As we expected, in normal culture condition PANC1 cells mainly rely on glucose as nutrition source for *de novo* lipogenesis, with negligible contribution from glutamine (Fig. S1).

### Increased lipogenesis in pancreatic cancer cells compared to normal epithelial cells

**Figure 4:**
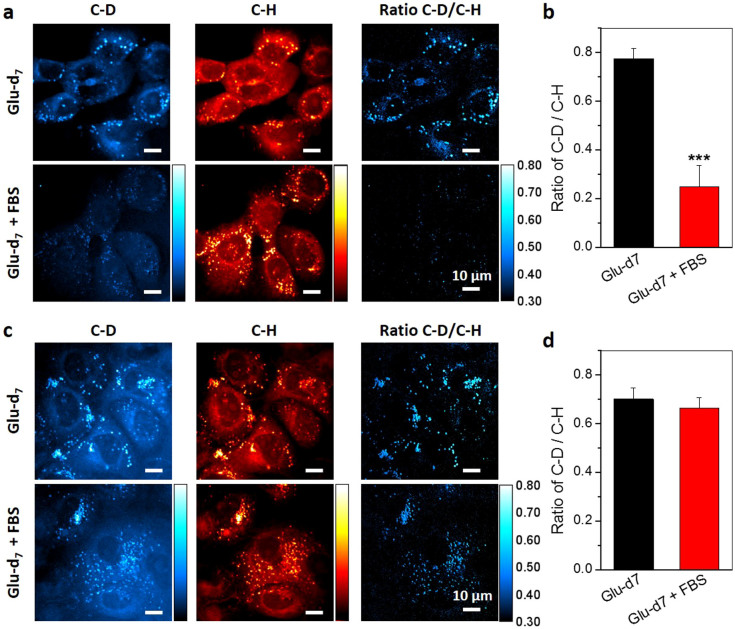
Increased lipogenesis in pancreatic cancer cells than normal pancreatic cells. (a). Normal immortalized pancreatic epithelial HPDE6 cells, and (c). pancreatic cancer PANC1 cells were treated with 25 mM glucose-d_7_ in glucose-free DMEM media supplemented without or with 10% FBS for 3 days. SRS imaging at C–D and C–H vibration were taken. The ratio of C–D/C–H was used to analyze the level of de-novo lipogenesis. Quantitative analysis of de-novo lipogenesis level in (b). HPDE6 and (d). PANC1 cells. The ratio of C–D signal to C–H signal intensity on single lipid droplets was measured. At least 10 lipid droplets were analyzed for each sample. The data was represented by Mean + SD. *** indicates p < 0.001 by student T test.

Increased lipogenesis is being accepted as an important player in cancer metabolism. Here, with glucose-d_7_ incubation, we compared the level of *de novo* lipogenesis in normal HPDE6 cell line (Fig. 4a) and two pancreatic cancer cell lines, PANC1 (Fig. 4b) and MIA PaCa2 (Fig. S2a). Generally, mammalian cells can acquire lipids from two sources, *de novo* lipogenesis or uptake from diet^4^. To evaluate the contribution of *de novo* lipogenesis and dietary uptake in normal and cancer cells, we incubated the cells with 25 mM gluose-d_7_ in glucose-free medium supplemented with or without 10% FBS for 3 days. The results (Fig. 4, S2) showed that without serum supplementation, both normal and cancerous cells utilize *de novo* lipogenesis and store the excess lipids in LDs. However, when the serum is supplemented, normal cells uptake exogenous lipids instead of employ *de novo* synthesis (Fig. 4a), whereas the pancreatic cancer cells use *de novo* synthesis regardless the presence of exogenous lipids (Fig. 4c, S2a). Based on the ratio of C–D signal over C–H signal, we showed that at presence of exogenous lipids, the *de novo* lipogenesis level in pancreatic cancer PANC1 (0.665 ± 0.042) and MIA PaCa2 cells (0.623 ± 0.026) is 2~3 folds higher than that in HPDE6 cells (0.249 ± 0.087) (Fig. 4b, 4d and Fig. S2b). Together, these results suggested the lipogenic pathway has been reprogrammed in pancreatic cancer cells compared to normal epithelial cells, which provides visual evidence for the previously reported up-regulation of *de novo* lipogenesis pathway in pancreatic cancer cells^27^,^28^.

### Lipogenesis in prostate cancer

**Figure 5:**
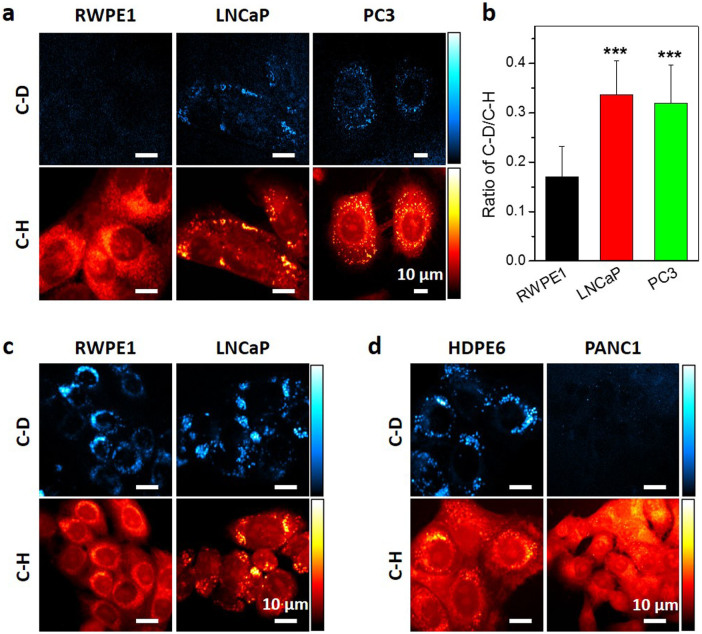
Lipogenesis and fatty acid uptake in prostate and pancreatic cells. (a). Glucose-d_7_ derived lipogenesis were measured in prostate cell lines, including normal epithelial RWPE1 cell and cancerous LNCaP, PC3 cells by SRS imaging at C–D and C–H vibration. (b). Quantitative analysis of de-novo lipogenesis level in prostate cell lines. Data were shown as mean + SD. N ≥ 10 for each group. *** indicates p < 0.001 by student T test. (c). SRS imaging of prostate cells and (d). pancreatic cells treated with 100 μM palmitic acid-d_31_ for 24 hours.

Though increased lipogenesis is accepted as a hallmark for cancers^29^, our live cell imaging study revealed that different cancer types might use distinct lipogenic pathways. With glucose-d_7_ and SRS imaging, we measured the lipogenesis in multiple types of cancer cells, including breast cancer MCF7, lung cancer A549, prostate cancer PC3 and pancreatic cancer PANC1 (Fig. S3a). Interestingly, we found that PC3 cells have lower level of *de novo* lipogenesis compared to other types of cancers (Fig. S3b), although the PC3 cells contained large amount of LDs. Considering PC3 is an androgen independent prostate cancer cell line^30^, to fully unveil the lipogenic profiles in prostate cancer we included normal epithelial RWPE1 cell, androgen dependent LNCaP and androgen independent PC3 cancer cell. Firstly, glucose-d7 was applied to prostate cells supplemented with 10% FBS. Our results showed that both LNCaP and PC3 cells exhibited elevated level of *de novo* lipogenesis than RWPE1 cells (Fig. 5a). The ratio of C–D signal to C–H signal is 0.336 ± 0.070 for LNCaP and 0.320 ± 0.078 for PC3 cell. Although they are much higher than RWPE1 (0.171 ± 0.062) cells, they are lower than pancreatic cancer PANC1 and MIA PaCa-2 cell (Fig. 5b, 4d and Fig. S2b). Furthermore, to investigate whether the fatty acid uptake contribute to the lipid accumulation in PC3 cells, deuterium labeled palmitic acid-d_31_ was used. Similarly, cells were incubated with palmitic acid-d_31_ for 24 hours and imaged at C–D vibration to visualize the deuterium labeled LDs derived from exogenous palmitic acid-d_31_. The results showed that under same condition, both RWPE1 and LNCaP cells uptake high level of free fatty acids (Fig. 5c). However, pancreatic cancer PANC1 cells have much reduced level of fatty acid uptake compared to normal HPDE6 cells (Fig. 5d). All together, these data suggest that prostate cancer cells, though with increased *de novo* lipogenesis, still remain the capability for high level of fatty acid uptake.

## Discussion

Visualizing metabolism in single living cells has been challenging due to the technical difficulties. By using deuterium labeled glucose and fatty acids, we have demonstrated the capability of SRS microscopy in imaging metabolic processes in temporal and spatial resolved manner. This method is well complementary to current techniques, like mass spectrometry or NMR spectroscopy. High spatial resolution, instead of ensemble measurement, is important for study of dynamic and complex metabolic networks. Currently, cancer heterogeneity in genetic aspect has been well accepted. However, heterogeneity in metabolism has not been well studied. Whether the variety in genetic mutations drives the metabolic heterogeneity or the diversity in metabolism affect the cancer heterogeneity remains an open question^31^,^32^. Our method would offer an avenue to address this question by imaging the metabolism in living cells or live tissues in situ. Another important question that can be pursued by our technology is whether there is a difference between cancer stem cell, progenitor cells, and regular cancer cells regarding to the metabolism aspect^33^,^34^. In general, the ability to visualize metabolism in single living cells would potentially be important to understanding cancer heterogeneity.

Besides imaging lipogenesis process, our method can potentially be used broadly on other aspects of metabolism. Isotope labeling is an ideal tracer for metabolism, especially for small molecules, because the labeling generally does not interfere their normal functions. Recently, isotope labeled amino acids have been demonstrated to monitor newly synthesized proteins^20^ and proteome degradation^35^. Choline and its metabolites in living systems have been imaged by SRS with deuterium labeled choline^36^. In addition, isotope labeling could also be applied to small molecules, like drugs. Currently, drug metabolism in single cell level is still not fully understood. A recent paper demonstrates SRS imaging resolves the distribution of anticancer drugs in living cells^37^. To trace the fate of isotope labeled drugs in single cells with subcellular resolution will be important to understand its metabolic pathway and further improve the efficiency of drug delivery.

In this work, we showed that compared to pancreatic cancer cells, prostate cancer cells exhibit lower level of *de novo* lipogenesis but higher level of fatty acid uptake. Our observation is consistent with previous report of dominant fatty acid uptake over glucose uptake in prostate cells^38^. The difference of lipogenic preference of prostate cancer may be related to its special metabolic demands. It has been reported that prostate cancer relies more on β-oxidation pathway as an energy source^39^. Increased β-oxidation process may require higher and faster demands of free fatty acids, which cannot be easily achieved by *de novo* lipogenesis. Our observation could be a result of this altered fatty acid metabolism pathway in prostate cancer. However, we are also aware of that different cell line model may have distinct metabolic profiles^40^. Further investigations are needed to fully understand the complexity of metabolic networks. Our imaging method could be a powerful tool to reveal the metabolic differences between different cell models in the future studies.

More applications of our method rely on further improvement of imaging sensitivity. Due to limited sensitivity at mM level, we could not detect other metabolites of glucose except lipids. Higher sensitivity would allow mapping the metabolic transformation by using glucose as precursor for other macromolecular synthesis, such as nucleotides and proteins. The other way to enhance the capability of this technique is to perform hyperspectral imaging instead of single-frequency imaging. As isotope-labeled molecules are converted to other metabolites, the vibration frequency of the C–D bond may shift due to environment change. Hyperspectral imaging would allow us to separate different metabolic species based on the spectral differences^41^. With higher detection sensitivity or spectral acquisition capability, SRS imaging would be fully appreciated for single cell metabolism study.

## Methods

### SRS microscopy and Raman spectroscopy

SRS microscopy was performed with two femto-second laser system, as described previously^23^. Specifically, a Ti:Sapphire laser (Chameleon Vision, Coherent) with up to 4 W (80 MHz, ~140 fs pulse width) pumps an optical parametric oscillator (OPO, Chameleon Compact, Angewandte Physik & Elektronik GmbH). The pump and Stokes beams were tuned to 830 nm and 1004 nm, respectively, to be in resonance with the C–D vibration at 2120 cm^−1^. For imaging at C–H vibration, pump and Stokes beams were tuned to 830 nm and 1090 nm, respectively. The pump and Stokes pulse trains were collinearly overlapped and directed into a laser-scanning microscope (FV300, Olympus). A 60X water-immersion objective lens (UPlanSApo, Olympus) was used to focus the laser into a sample. An oil condenser of 1.4 numerical aperture (NA) was used to collect the signal in a forward direction. The typical acquisition time for a 512 × 512 pixels SRL image was 1.12 second. Because most excitation power was carried by the Stokes beam at 1090 nm, with the pump beam power being as low as a few mW at the sample, photodamage to cells was not detected.

For spontaneous Raman spectroscopy, a 5-ps Ti:sapphire laser (Tsunami, Spectra-Physics, Mountain View, CA) tuned to ~707 nm was used^42^. Each Raman spectrum was acquired in 20 seconds, and pump laser power at the specimen was maintained at 15 mW.

### Chemicals and Reagents

Glucose-d_7_, glutamine-d_5_ and palmitic acid-d_31_ were purchased from Cambridge Isotope Laboratories, Inc. Fatty acid synthase (FAS) inhibitor C75 was purchased from Cayman Chemical. 2-deoxy-D-glucose (2-DG) was purchased from Sigma (Cat# D8375). High glucose (4.5 g/ml), glucose free and glucose, glutamine free DMEM medium, RPMI 1640, Keratinocyte Serum Free Medium (K-SFM) with additives bovine pituitary extract (BPE) and human recombinant epidermal growth factor (rEGF) were purchased from Invitrogen Life Technologies. F-12K medium was purchased from ATCC. Fetal bovine serum (FBS) was purchased from Sigma.

### Cell culture and imaging

All cells were cultured in 37°C moisture incubator with 5% CO_2_ supply. The following media were used for maintenance: DMEM high glucose medium supplemented with 10% FBS was used for PANC1 and A549 cell; RPMI 1640 supplemented with 10% FBS was used for MIA PaCa2, MCF7 and LNCaP cell; F-12K supplemented with 10% FBS was used for PC3 cell; K-SFM supplemented with 30 μg/ml BPE and 0.2 ng/ml rEGF was used for HPDE6 and RWPE1 cell. For SRS imaging of glucose-d_7_, around 0.3 M cells were seeded on glass-bottom dishes (In Vitro Scientific, Cat# D35-10-1.5-N) with 25 mM glucose-d_7_ in glucose-free DMEM media supplemented with or without 10% FBS. For the study of glutamine metabolism, the cells were incubated with 25 mM glucose-d_7_ and 4 mM glutamine-d_5_ in glucose, glutamine-free DMEM media supplemented with 10% FBS for 3 days. For the fatty acid uptake study, cells were incubated with 100 μM palmitic acid-d_31_ in DMEM high glucose media without 10% FBS for 24 hours. For cell dynamic study, living cells were used. For quantitative studies, cells were fixed in 10% neutral buffered formalin for 30 min.

### Transfection of FAS shRNA

FAS shRNA plasmid was purchased from Santa Cruz (Cat# sc-29311-SH). Transfection of shRNA plasmid to PANC1 cells was performed using Lipofectamine® 2000 (Invitrogen, Cat# 11668-019) by following the manufacturer's protocol. Stable transfected colony was selected under 2 μg/ml puromycin for 10 days.

### Cell toxicity test

Cells were seeded in 96-well plates (~5000 cells/well) and grown overnight. Then the medium was replaced with 25 mM glucose or glucose-d7 in glucose-free DMEM media supplemented with 10% FBS for 1~3 days. Cell viability was measured with the MTT colorimetric assay (Sigma) every day after treatment.

### Data analysis

Quantification of LDs was analyzed with ImageJ software particle analysis function. Area fraction was used to present the amount of LDs in a single cell. For analysis of the C–D/C–H ratio, threshold was firstly applied to the C–D image to pick up the LDs. Then the ratio was obtained by dividing C–D images over C–H images. Statistical analysis was done with the C–D/C–H ratio of single LD.

## Author Contributions

J.L. and J.X.C. conceived and designed the methods and experiments. J.L. conducted the experiments and data analysis. J.L. and J.X.C. wrote the manuscript.

## Supplementary Material

Supplementary Information Supplementary information

Supplementary Information Movie
