# Supplementary material for: Direct Visualization of De novo Lipogenesis in Single Living Cells
Source: Sci Rep. 2014 Oct 29;4:6807. doi: 10.1038/srep06807 (PMC4212242; doi:10.1038/srep06807)
Supplement: Supplementary Information [file srep06807-s1.pdf]

Supplementary information

Direct Visualization of De-novo Lipogenesis in Single Living Cells

Junjie Li[1, 2], Ji-Xin Cheng[1, 3*]

[1] Weldon School of Biomedical Engineering, Purdue University, West Lafayette, IN 47907

[2] Department of Biological Sciences, Purdue University, West Lafayette, IN 47907

[3] Center for Cancer Research, Purdue University, West Lafayette, IN 47907

* Corresponding author: jcheng@purdue.edu, 765-494-4335

Supplementary figure captions.

Movie showing the dynamic motions of newly synthesized lipid droplets in living cells.

Supplementary figure 1: Lipogenesis derived from glucose-d_7 or glutamine-d_5 metabolic pathway. PANC1 cells were treated with 25 mM glucose-d_7 or 4 mM glutamine-d5 or both in glucose, glutamine-free DMEM media for 3 days. SRS imaging at C-D and C-H vibration were taken.

Supplementary figure 2: Lipogenesis in pancreatic cancer MIA PaCa2 cells. a. MIA PaCa2 cells were treated with 25 mM glucose-d_7 in glucose-free DMEM media supplemented without or with 10% FBS for 3 days. SRS imaging at C-D and C-H vibration were taken. The ratio of C-D/C-H was used to analyze the level of de-novo lipogenesis. **b.** Quantitative analysis of de-novo lipogenesis level in MIA PaCa2 cells. The ratio of C-D signal to C-H signal intensity on single LD was measured. Totally 10 lipid droplets were analyzed for each sample. The data was represented by Mean + SD.

Supplementary figure 3: Lipogenesis and lipid uptake in different cancer cell lines. a. Glucose-d_7 derived lipogenesis were measured in different cancer cell lines, including breast cancer MCF7, lung cancer A549, prostate cancer PC3 and pancreatic cancer PANC1 by SRS imaging at C-D and C-H vibration. **b.** Quantification of SRS signal at C-D vibration per cell. Data were shown as mean + SD. N= 5 for each group.

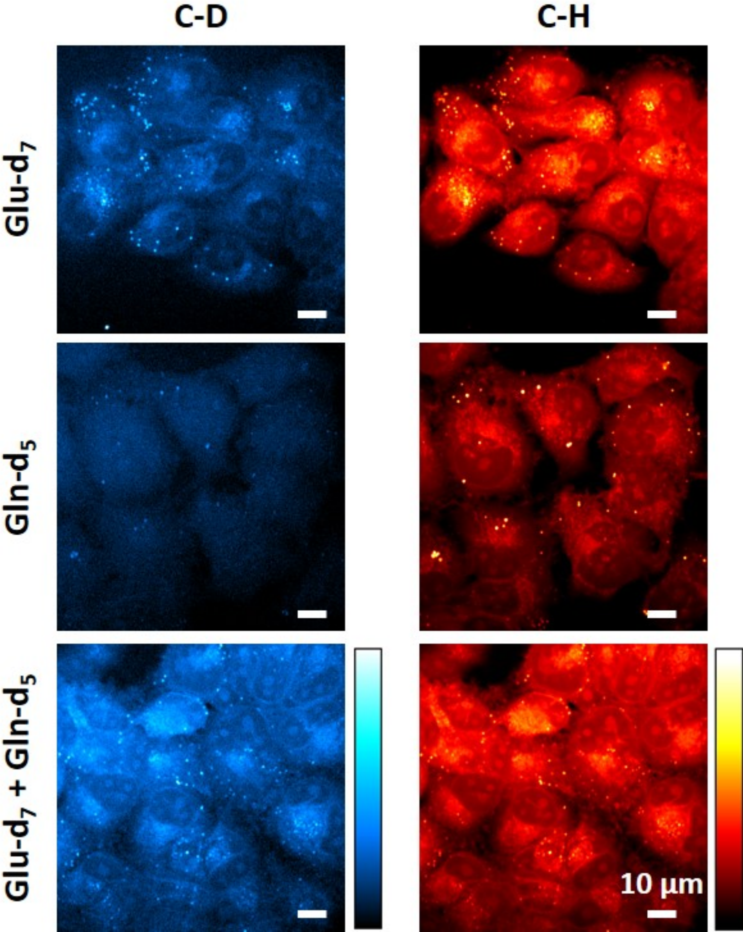

C-D
C-H
Glu-d7
Gln-d5
Glu-d7 + Gln-d5
10 µm

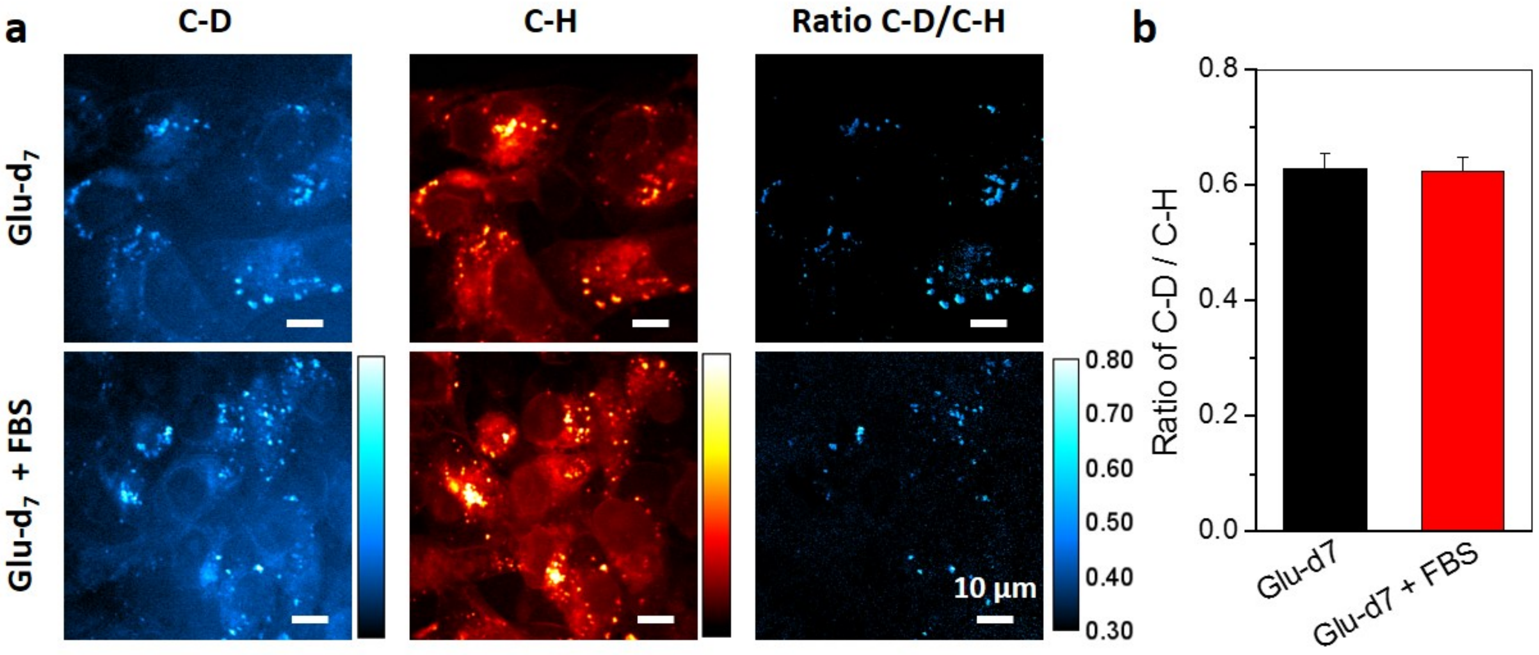

a
C-D
C-H
Ratio C-D/C-H
Glu-d7
Glu-d7 + FBS
10 μm
0.80
0.70
0.60
0.50
0.40
0.30
b
Ratio of C-D / C-H
0.0
0.2
0.4
0.6
0.8
Glu-d7
Glu-d7 + FBS

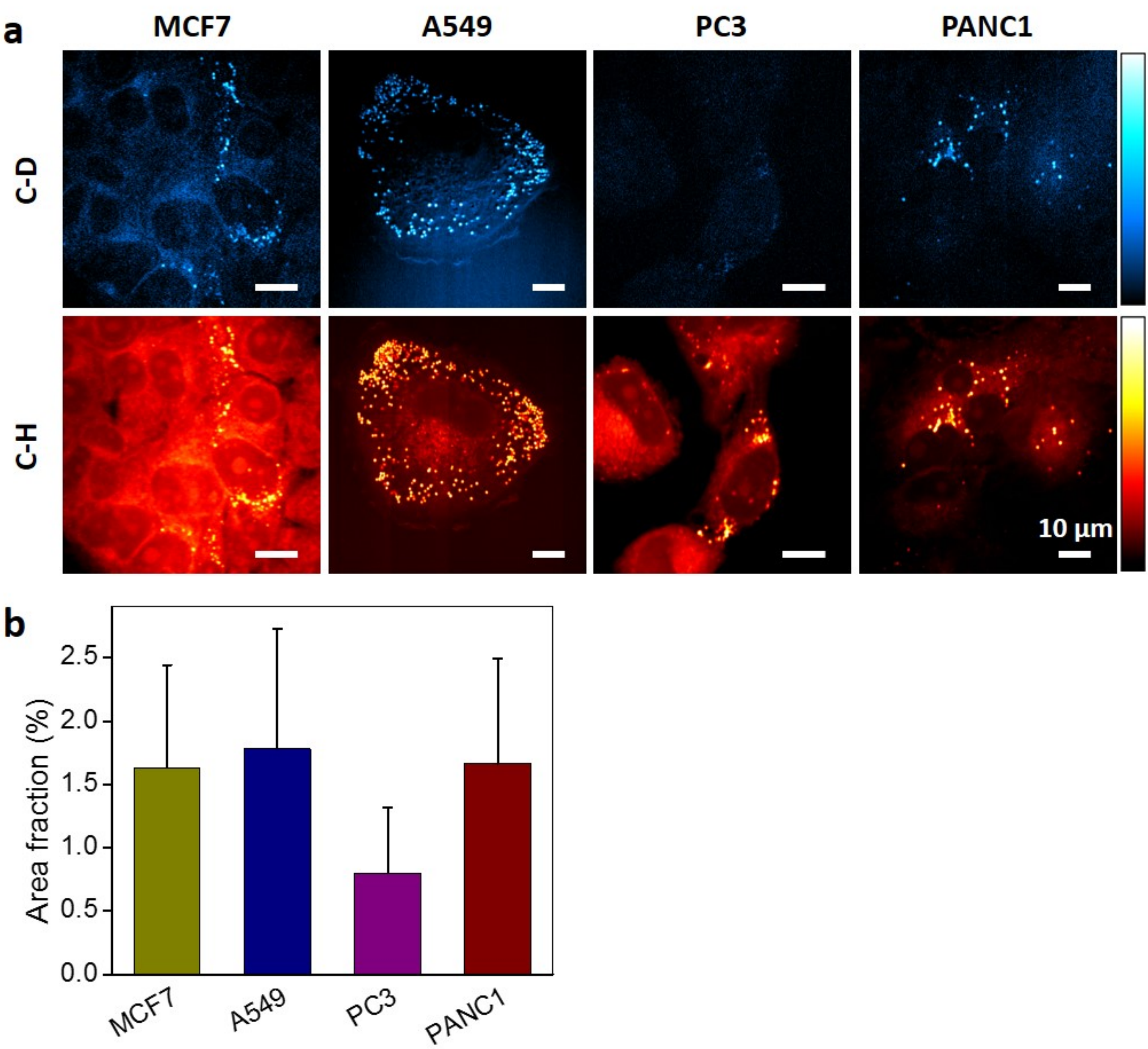

a
MCF7
A549
PC3
PANC1
C-D
C-H
10 µm
b
Area fraction (%)
0.0
0.5
1.0
1.5
2.0
2.5
MCF7
A549
PC3
PANC1
